# Left ventricular torsion shear angle volume analysis in patients with hypertension: a global approach for LV diastolic function

**DOI:** 10.1186/s12968-014-0070-4

**Published:** 2014-09-26

**Authors:** Chun G Schiros, Ravi V Desai, Bharath Ambale Venkatesh, Krishna K Gaddam, Shilpi Agarwal, Steven G Lloyd, David A Calhoun, Thomas S Denney, Louis J Dell’italia, Himanshu Gupta

**Affiliations:** Division of Cardiovascular Medicine, Department of Medicine, University of Alabama, BDB 101, CVMRI, 1530 3rd Ave South, Birmingham, AL 35294-0012 USA; Johns Hopkins Hospital, Baltimore, MD USA; Birmingham Veteran Affairs Medical Center, Birmingham, AL USA; Department of Electrical and Computer Engineering, Auburn University, Auburn, AL USA

**Keywords:** Torsion hysteresis area, $\widehat{\varphi}\widehat{V}$ loop, Diastolic function, Cardiovascular magnetic resonance

## Abstract

**Background:**

Torsion shear angle *φ* is an important measure of left ventricular (LV) systolic and diastolic functions. Here we provide a novel index utilizing LV normalized torsion shear angle $\left(\widehat{\varphi}\right)$ volume $\left(\widehat{V}\right)$ loop to assess LV diastolic functional properties. We defined the area within $\widehat{\varphi}\widehat{V}$ loop as torsion hysteresis area, and hypothesized that it may be an important global parameter of diastolic function. We evaluated the $\widehat{\varphi}$ changes to increased $\widehat{V}$ during early diastole $\left(-d\widehat{\varphi}/d\widehat{V}\right)$ as a potential measure of LV suction.

**Methods:**

Sixty resistant hypertension patients (HTN), forty control volunteers were studied using cardiovascular magnetic resonance with tissue tagging. Volumetric and torsional parameters were evaluated.

**Results:**

HTN demonstrated concentric remodeling with preserved ejection fraction. HTN had significantly decreased normalized early filling rate, early diastolic mitral annulus velocity and E/A (1.33 ± 1.13 vs. 2.19 ± 1.07, P < 0.0001) vs. control. Torsion hysteresis area was greater (0.11 ± 0.07 vs. 0.079 ± 0.045, P < 0.001) and peak $-d\widehat{\varphi}/d\widehat{V}$ at early diastole was higher (10.46 ± 8.51 vs. 6.29 ± 3.85, P = 0.002) than control. Torsion hysteresis area was significantly correlated with E/A (r = −0.23, P = 0.025). Thirteen HTN patients had both E/A ratio < 1.12 (Control mean E/A-1SD) and torsion hysteresis area > 0.12 (Control mean torsion hysteresis area + 1SD).

**Conclusions:**

Torsion hysteresis area and peak early diastolic $-d\widehat{\varphi}/d\widehat{V}$ were significantly increased in hypertensive concentric remodeling. The $\widehat{\varphi}\widehat{V}$ loop takes into account the active and passive recoil processes of LV diastolic and systolic phases, therefore provides a new global description of LV diastolic function.

## Background

Left ventricular (LV) diastolic dysfunction is characterized by abnormal myocardial mechanical properties that include impaired diastolic distensibility, impaired LV filling and slow or delayed myocardial relaxation [1]. Up to 50% of patients with heart failure have predominant diastolic dysfunction in the presence of preserved LV ejection fraction (EF) [2].

Invasive hemodynamic measurements, such as the time constant of relaxation(1), τ, and LV end diastolic pressure volume relationship (EDPVR) [3] are considered useful to assess diastolic function. However, they are not frequently performed in routine clinical practice. In contrast, echocardiographic tissue Doppler measurement of early diastolic mitral annular velocity with other appropriate parameters is frequently used as a non-invasive diagnostic tool of diastolic dysfunction [4]. However these methodologies may have significant limitations [5].

LV twist, measured as the myocardial rotation gradient from the base to apex along a longitudinal axis [6], is an important mechanical property of the myocardium that results from the helical fiber arrangement of LV. Torsion shear angle is twist normalized to long-axis length and LV radius [6]. Contraction of myocardial bundles and their interaction with extracellular matrix during systole result in storage of torsional potential energy. Torsional recoil during isovolumic relaxation and early diastole releases the potential energy stored in the deformed matrix during systole [7,8]. Previous work with cardiovascular magnetic resonance (CMR) in an animal model [9] and recently in humans [10], showed that LV early untwist rate correlated closely with τ.

In this paper, a global approach utilizing normalized LV torsion shear angle volume (normalized *φ* is indicated as $\widehat{\varphi}$ and normalized *V* is indicated as $\widehat{V}$) loop is proposed to assess LV diastolic function. Torsion hysteresis area (THA) is quantified as the area within the $\widehat{\varphi}\widehat{V}$ loop. This ‘hysteresis’ concept was inspired by the history-dependent stress strain relationships in viscoelastic materials undergoing cyclic loading, representing energy loss or work. As it is difficult to measure instantaneous LV wall stress non-invasively, temporal changes in LV volume throughout the cardiac cycle provide an indirect measure of changes in ventricular stress. Moreover, LV wall tensile stress is directly related to wall geometry and wall thickness. Torsion shear angle is also affected by these factors, representing a global measure of ventricular strain. We therefore evaluated the relationship of changes in torsion shear angle to LV volumetric changes over the cardiac cycle and develop THA as a non-invasive measurement which quantifies the relationship between myocardial strain and volume change. Normalizing torsion shear angle and volume allow for comparison among groups with various geometry remodeling. Furthermore, since untwist is an important mechanism of LV suction, we explored the relationship of peak $\widehat{\varphi}$ changes to increase in $\widehat{V}$ during early diastolic phase $\left(-d\widehat{\varphi}/d\widehat{V}\right)$ as a potential measure of LV suction.

We hypothesized that $\widehat{\varphi}\widehat{V}$ loop represented a new global approach to assess diastolic function. THA and peak $-d\widehat{\varphi}/d\widehat{V}$ at the early diastole derived from the loop are higher in patients with resistant hypertension (HTN).

## Methods

### Study population

The study population consisted of 40 normal control volunteers and 60 HTN, defined as uncontrolled hypertension (ambulatory BP > 140/90 mmHg at two or more clinic visits in spite of the use of three or more antihypertensive medications at optimal doses). Detailed methodology for enrollment of HTN patients in this prospective study design was previously described [11]. The normal control volunteers had no history of cardiovascular disease and were not taking any cardiovascular medicines. Cine and tagged CMR was performed on all HTN patients and control volunteers for comparison purposes. Early morning ambulatory brain natriuretic peptide (BNP) was measured in HTN. The study protocol was approved by the Institutional Review Boards of University of Alabama at Birmingham and Auburn University and informed consent was obtained from all participants.

### Cardiovascular Magnetic Resonance (CMR)

Cine CMR was performed on a 1.5-T MRI scanner (Signa, GE, Milwaukee, Wisconsin) optimized for cardiac application. Electrocardiographically gated breath-hold steady-state free precision technique was used to obtain standard (2-, 3-, and 4-chamber long axis and short-axis) views using the following general parameters: slice thickness of the imaging planes 8 mm, field of view 40 × 40 cm, scan matrix 256 × 128, flip angle 45°, repetition/echo times 3.8/1.6 ms, views per segment 8–10, number of reconstructed cardiac phases 20.

Tagged CMR was done on exact slice prescriptions as above by applying grid tagging to the short axis views and stripe tagging to long axis views using spatial modulation of magnetization encoding gradients method (FGR-SPAMM) as previously described [12] with following general parameters: prospective ECG triggering, trigger time 10 ms from R wave, slice thickness 8 mm, zero interslice gap, field of view 40 × 40 cm, scan matrix 256 × 128, flip angle 10°, repetition/echo times 8.0/4.2 ms, views per segment 8–10, tag spacing 7 mm and number of reconstructed cardiac phases 20. Because the tag lines faded with time due to T_1_ relaxation, tagged image derived parameters were only valid throughout systole and the first 67% of diastole.

LV geometric parameters were measured from endocardial and epicardial contours manually traced on cine images acquired near end-diastole (ED) and end-systole (ES). These contours were propagated throughout the cardiac cycle using in-house software [13]. LV volume, volume time curve and its derived peak ejection rates, peak early and late filling rates were calculated as described [13]. LVED mass was measured excluding the papillary muscle. Peak early and late diastolic mitral annular velocities were calculated using non-rigid registration to track a manually-selected point on the mitral annulus through the cardiac cycle [13]. The 3D radius of curvature to wall thickness ratio (R/T) was computed by the reciprocal of the product of the endocardial circumferential curvature and 3D wall thickness as previously described [14].

Two-dimensional (2D) strain at each timeframe and rates were measured using harmonic phase (HARP) analysis [15]. 2D basal and apical rotations at each timeframe were measured by tracking a circular mesh of points in the basal and apical slice of that timeframe. The mesh was identified in the first time based on user-defined contours and tracked through the remaining imaged phases using improved HARP tracking [16]. 2D twist was computed as the apical rotation minus the basal rotation at the same timeframe. Twist time curve was constructed and differentiated to obtain the twist rate time curve. Peak early diastolic untwist rate was defined as the maximum twist rate at the early diastolic phase. Torsion shear angle *φ* at timeframe *t* was computed as [6]

$$ \varphi (t)=T(t)\times \frac{\rho_{base}(t)+{\rho}_{apex}(t)}{2L}; $$

where *ρ(t)* is the epicardial radius at time *t* and *L* is the distance between the basal and distal slices at ED timeframe. 2D *φt* curve was therefore constructed for each subject.

### Torsion shear angle volume loop

**Figure 1:**
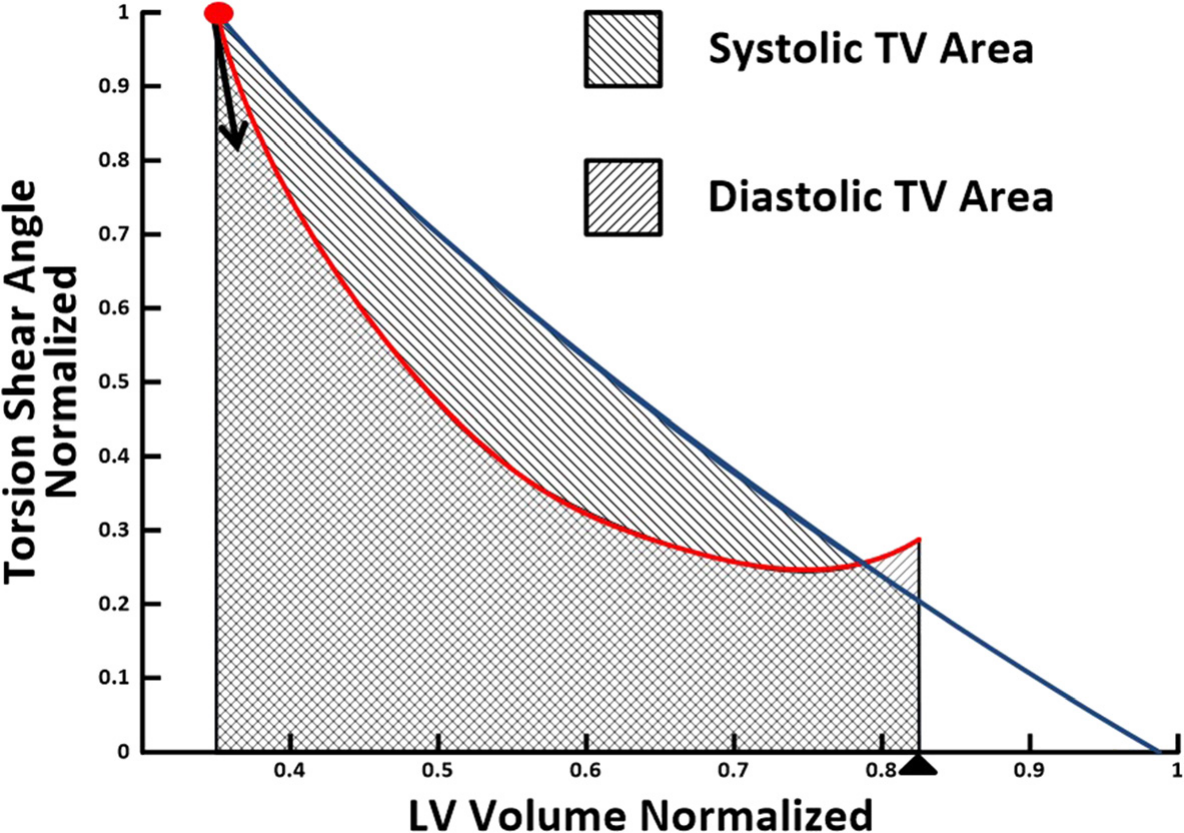
**Schematic diagram for calculating torsion hysteresis area.** Torsion hysteresis area represents the area within the systolic and diastolic arm of the normalized torsion shear angle-volume $\left(\widehat{\varphi}\widehat{V}\right)$ curves. Red curve, diastolic arm; blue curve, systolic arm; red dot, end systole mark; black triangle, 67% of diastole mark; black downward arrow indicates the peak $-d\widehat{\varphi}/d\widehat{V}$ at early diastole.

For each subject, the *φt* curve was normalized by its maximum to generate $\widehat{\varphi}t$ curve and LV *Vt* curve was normalized by its maximum *V* to generate $\widehat{V}t$ curve. Then by forcing the maximum normalized torsion shear angel $\widehat{\varphi}$ and minimum normalized volume $\widehat{V}$ of each subject to be at the same time point, the systolic and diastolic phases of the $\widehat{\varphi}$*t* curve and $\widehat{V}$*t* curve were interpolated with 10 and 18 time points respectively to create the $\widehat{\varphi}\widehat{V}$ loop as shown in Figure 1. This process was called ‘extrema matching’ and it was implemented to account for the differences in temporal resolution and changes in heart rate in cine vs tag MR images. As MR tag lines fade during mid to late diastole and torsion shear angle cannot be reliably measured, for consistency, we chose the first 12 interpolated data points (67% of diastole) in diastole for torsion hysteresis area computation. Area under the systolic and diastolic arms of the $\widehat{\varphi}\widehat{V}$ curve were computed numerically using the trapezoidal rule (Figure 1). Systolic and diastolic areas were computed over the volume interval between ES and 67% diastole. THA was computed by subtracting the diastolic area from the systolic area. Peak $-d\widehat{\varphi}/d\widehat{V}$ at the early diastole phase was calculated as the negative peak slope of the diastolic arm of the $\widehat{\varphi}\widehat{V}$ curve. The peak slope of the diastolic arm of the $\widehat{\varphi}\widehat{V}$ curve was defined as the slope of a fitted linear regression model to the first four points of the diastolic arm. Both THA and Peak $-d\widehat{\varphi}/d\widehat{V}$ were computed for each subject and averaged over each group.

### Statistical analysis

Student’s two-sample *t* test (for continuous variables) and Fisher’s exact test (for categorical variables) were conducted to compare control and HTN in terms of demographic, geometric, and functional variables. Pearson’s correlation analysis was THA and other CMR-derived indices of LV diastolic function (normalized peak early filling rate, E/A ratio, normalized peak early diastolic mitral annulus velocity) as well as LV mass/volume ratio and mean atrial pressure (MAP). We also created multivariate model for torsional and strain rate parameters to adjust for age using linear regression models.

Data are in mean ± standard deviation (SD). A P < 0.05 was considered statistically significant. All statistical analysis was performed using SAS version 9.3.

## Results

### Patient demographics

Control’s mean age was significantly lower than that of HTN (Table 1). Thus, the comparisons between control and HTN in torsional and strain parameters were adjusted for age. HTN had higher systolic and diastolic blood pressures vs. controls at the time of CMR. BNP level in HTN was measured as 35.25 ± 34.32 pg/mL, ranging from 2 to 169.8 pg/mL.

**Table 1:** **Basic characteristics**

	**Control**	**HTN**
	**(n = 40)**	**(n = 60)**
**Age, years**	42 ± 13	55 ± 12*
**Age range, years**	20-69	26-76
**Males,%**	52	52
**SBP, mmHg**	118 ± 14	144 ± 16*
**DBP, mmHg**	74 ± 11	88 ± 13*
**HR, beats/minute**	71 ± 13	68 ± 12
**Duration of HTN, years**	0	17 ± 10
**Number of anti-HTN medications**	0	4 ± 1
**Beta blocker (%)**	0	75
**ACE-I (%)**	0	62
**ARB (%)**	0	53
**CCB (%)**	0	68
**Diuretic (%)**	0	92
**Other (%)**	0	37
**BUN, mg/dl**	11.73 ± 3.69	13.04 ± 6.19
**Creatinine, mg/dl**	0.94 ± 0.19	1.05 ± 0.26*

Values are n or mean ± SD; HTN: hypertension; BSA: body surface area; SBP: systolic blood pressure measured at the time of CMR; DBP: diastolic blood pressure measured at the time of CMR; HR: heart rate; ACE-I: Angiotensin-converting enzyme inhibitor; ARB: Angiotensin receptor blocker; CCB: Calcium channel blocker; BUN: Blood urea nitrogen; * = significantly different from Control.

### CMR-derived LV geometric and systolic functional parameters

Controls and HTN did not differ in volumes and stroke volume indices (normalized to BSA, as shown in Table 2). LV mass and LV mass/volume ratio were significantly increased while LVED R/T ratio was significantly decreased in HTN vs. controls. Systolic function measured as LVEF and normalized peak ejection rate were higher in HTN.

**Table 2:** **CMR-derived volumetric data**

	**Control**	**HTN**
	**(n = 40)**	**(n = 60)**
**LVED Volume index, ml/m** ^**2**^	71 ± 15	72 ± 22
**LVES Volume index, ml/m** ^**2**^	25 ± 7	22 ± 10
**LV Stroke volume index, ml/m** ^**2**^	46 ± 10	49 ± 14
**LVEF, %**	65 ± 6	70 ± 8*
**Peak Ejection rate, EDV/s**	3.11 ± 0.44	3.45 ± 0.68*
**LV Mass Index, grams/height** ^**2.7**^	22 ± 7	32 ± 13*
**LV Mass/Volume, grams/ml**	0.76 ± 0.17	0.98 ± 0.26*
**LVED R/T**	3.76 ± 0.87	3.05 ± 0.76*

Values are mean ± SD; HTN: hypertension; ED: end-diastole; ES: end-systole; EF: ejection fraction; R/T: radius to wall thickness; Volume index: volume normalized to body surface area; Mass index: mass normalized the height^2.7^ in m; *: significantly different from Control.

### CMR-derived LV diastolic functional parameters

HTN demonstrated decreased CMR-derived volumetric peak early filling rate and greater peak late filling rate expressed as EDV/s, and lower E/A ratio (1.33 ± 1.13 vs. 2.19 ± 1.07, P < 0.0001) vs. controls (Table 3). Moreover, HTN depicted lower peak early diastolic mitral annular velocity and higher peak late diastolic mitral annular velocity vs. controls. Peak early diastolic circumferential and longitudinal strain rates were significantly lower in HTN vs. controls. Adjusted for age, these differences were still significant. Peak untwist rate in the early diastole was significantly increased in HTN with and without adjusted for age. Time to peak early untwist rate did not differ in HTN vs. controls.

**Table 3:** **CMR derived diastolic function parameters**

	**Control**	**HTN**
	**(n = 40)**	**(n = 60)**
**Normalized peak early filling rate, 1/s**	3.03 ± 0.60	2.54 ± 0.72*
**Normalized peak atrial filling rate, 1/s**	1.66 ± 0.65	2.32 ± 0.83*
**E/A Ratio**	2.19 ± 1.07	1.33 ± 1.13*
**Normalized peak E Dia MA velocity, %/sec**	86 ± 30	66 ± 21*
**Normalized peak A Dia MA velocity, %/sec**	43 ± 21	58 ± 23*
**Peak E Dia Circ. strain rate, %/sec†**	101 ± 28	79 ± 27*
**Peak E Dia long. strain rate, %/sec†**	104 ± 32	79 ± 30*
**Peak E Dia untwist rate, °/sec†**	78 ± 21	103 ± 39*
**Time to peak untwist rate, msec**	384 ± 42	398 ± 51

Values are mean ± SD; HTN: hypertension; E Dia: early diastole; A Dia: atrial diastole; MA: mitral annulus; Circ.: circumferential; Long.: longitudinal; * = significantly different from Control; †, the statistical significance retains after adjusting for age effect. Peak early and atrial filling rates were normalized to end-diastolic volume. Peak early and atrial diastolic MA velocity were normalized to left ventricular length multiplied by 100.

### LV torsion shear angle volume loop

**Figure 2:**
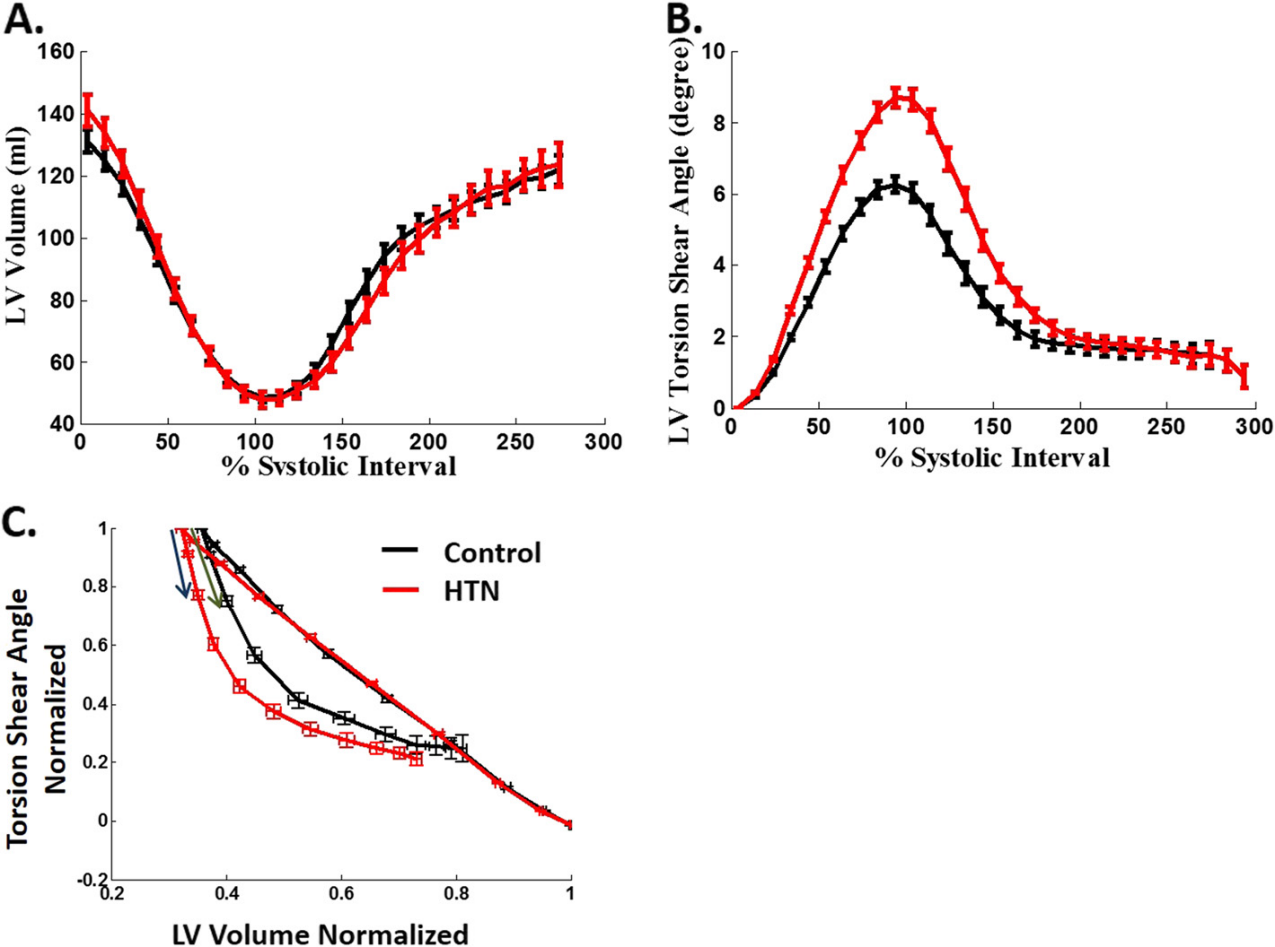
**Volume-time curves (A), torsion shear angle-time curves (B) and torsion shear angle-volume** $\left(\widehat{\boldsymbol{\upvarphi}}\widehat{\mathbf{V}}\right)$ **curves (C) for the HTN and control groups.** Data are mean ± SE. Early filling rate is significantly decreased in HTN with a higher peak systolic torsion shear angle compared with controls. HTN $\widehat{\varphi}\widehat{V}$ curve slightly shifts to the left of the control $\widehat{\varphi}\widehat{V}$ curve with a steeper early diastolic arm (indicated by blue arrow vs. green arrow) vs. controls.

The volume vs. time curve (Figure 2A) indicates an increase in peak ejection rate (Table 2) and a decrease in early filling rate (Table 3); while the torsion shear angle vs. time curve (Figure 2B) indicates an increase of peak torsion shear angle (9.18 ± 2.33 vs. 6.72 ± 1.66°, P < 0.0001) in the HTN group vs. controls. HTN $\widehat{\varphi}\widehat{V}$ curve (Figure 2C) slightly shifted to the left of the control $\widehat{\varphi}\widehat{V}$ loop with a steeper diastolic arm. THA and peak $-d\widehat{\varphi}/d\widehat{V}$ at the early diastole phase were significantly increased in HTN vs. controls (Table 4). After adjusted for age, these significant differences still persisted.

**Table 4:** **Torsion shear angle-volume loop derived parameters**

	**Control**	**HTN**
	**(n = 40)**	**(n = 60)**
**Torsion hysteresis†**	0.078 ± 0.045	0.11 ± 0.067*
**Peak E Dia** $-\mathbf{d}\widehat{\boldsymbol{\upvarphi}}/\mathbf{d}\widehat{\mathbf{V}}$ **†**	6.29 ± 3.85	10.45 ± 8.51*

Values are mean ± SD; HTN: hypertension; E Dia: early diastole; $\widehat{\varphi}$: normalized torsion shear angle; $\widehat{V}$, normalized volume; * = significantly different from Control; †, the statistical significance retains after adjusting for age effect.

**Figure 3:**
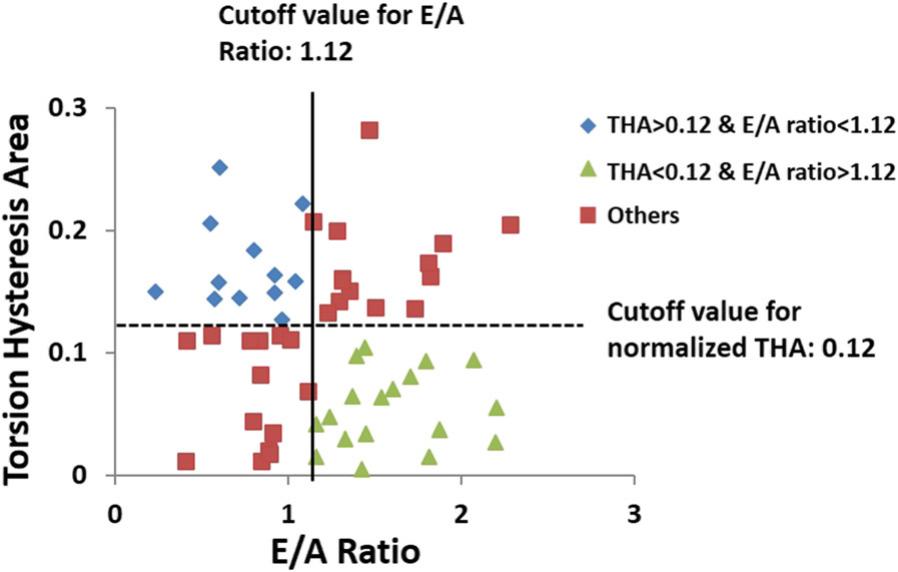
**Classification of HTN by E/A ratio and by torsion hysteresis area.** Cutoff values are defined as control’s mean-1SD for E/A ratio and control’s mean + 1SD for THA. Solid black line, cutoff value for E/A ratio; dash black line, cutoff value for THA; THA, torsion hysteresis area.

THA demonstrated significant correlation with E/A ratio (r = −0.23, P = 0.025). Twenty two percent HTN patients (13 out of 60) had both E/A ratio < 1.12 (Control mean E/A-1SD) and THA > 0.12 (Control mean THA + 1SD) as shown in Figure 3. Furthermore, THA was not significantly correlated with LV mass/volume ratio nor MAP.

## Discussion

This study provides a novel approach utilizing normalized torsion shear angle volume $\left(\widehat{\varphi}\widehat{V}\right)$ loop, which takes into account the global cardiac cycle, to assess diastolic function. We demonstrate, 1) the phenomenon of hysteresis, based on $\widehat{\varphi}\widehat{V}$ loop where the systolic component is not identical to the diastolic component of the loop; 2) that the $\widehat{\varphi}\widehat{V}$ loop derived THA is increased in HTN vs. controls; 3) HTN has increased peak early diastolic $-d\widehat{\varphi}/d\widehat{V}$ vs. control; 4) THA is significantly correlated with E/A ratio, a conventional parameter for diastolic function. These findings suggest stiffer ventricles with impaired suction and LV early filling in HTN. Therefore, $\widehat{\varphi}\widehat{V}$ loop may be a useful approach of non-invasive global assessment of diastolic function that takes into account not only active and passive recoil processes of the LV diastolic phase but also the systolic phase.

HTN is one of the most important risk factors for developing diastolic dysfunction and heart failure. This is attributed to maladaptive concentric LV remodeling, disorganization of sarcomeres, alteration in calcium handling, and increase in extracellular matrix [17]. Our HTN demonstrates concentric LV remodeling by decreased LVED R/T ratio and increased LV mass/volume ratio. Due to increased LV wall thickness with relatively greater epicardial radius compared to endocardial radius and other factors associated with the remodeling, there is likely a greater gradient of torque from epicardium to the endocardium in HTN vs. controls, resulting in a higher peak torsion shear angle in HTN that will therefore be associated with greater stored potential energy in the myocardium at ES. During diastole, some of the stored potential energy is used as work against the viscoelastic properties of myocardium and the rest likely contributes to LV filling and suction, myocardial relaxation and enhanced early diastolic filling [8,9,18].

In the current study, we find that peak early diastolic $-d\widehat{\varphi}/d\widehat{V}$ is significantly increased in HTN, indicating reduced LV filling to the same changes of untwist in HTN vs. controls. This therefore indicates impaired LV suction and early filling in HTN. Moreover, we find significantly higher THA in HTN. Therefore the abnormal torsion shear angle volume relationship obtained by changes in torsion shear angle to LV volumetric changes over the cardiac cycle in HTN group likely indicates inefficiency in ventricular relaxation due to stiffer/relatively non-compliant ventricle. This is further supported by reduced early diastolic strain rates in HTN vs. controls.

A number of echocardiographic parameters have been proposed for evaluating diastolic function [19], such as normalized peak early filling rate, E/A ratio, normalized peak early diastolic mitral annulus velocity. In our study, THA is significantly correlated with CMR-derived E/A ratio. Twenty-six percent HTN patients (13 out of 60) had both E/A ratio < 1.12 (control’s mean E/A-1SD) and THA > 0.12 (control’s mean THA + 1SD) as shown in Figure 3. HTN patients with small E/A ratio are not highly overlapped with patients with large THA, indicating that other factors may play an important role in determining THA, which is not expressed in E/A ratio. It is now increasingly recognized that patients with preserved ejection fraction and diastolic dysfunction may demonstrate impaired systolic strains at rest or blunted systolic response with exertion. Early diastolic E/A ratio is purely a diastolic filling parameter. The $\widehat{\varphi}\widehat{V}$ loop framework we propose is unique in that it takes into account both the systolic and diastolic phases and provides insight into ventricular stiffness and suction which are important determinants of diastolic dysfunction.

Previous study by Takeuchi M, et al. reported decreased early diastolic LV untwisting rate during isovolumic relaxation period in hypertensive patients utilizing 2D speckle tracking imaging [20]. Due to the relative lower temporal resolution of tagged CMR, peak untwist rate during isovolumic relaxation period is not available in our study. Instead, peak untwist rate during the early diastole phase is computed. However, in our study, we used widely available tagged CMR technique to define a new approach that wasn’t likely affected by the somewhat lower temporal resolution compared to echocardiography. MR tag lines faded during mid to late diastole and torsion shear angle cannot be reliably measured. In our study, we were able to evaluate 2/3 of the diastolic phase for all subjects. Since major portion of diastolic untwist happened quickly during early-diastolic phase and minimal untwisting occurred during late diastole, substantial change in current conclusion due to the limitation of our technique was unlikely. Regardless, our data demonstrates consistent differences amongst the two groups in the conventional non-invasive parameters frequently used for evaluating diastolic function.

## Conclusions

In conclusion, $\widehat{\varphi}\widehat{V}$ loop may be an important global approach to assess diastolic function. To our knowledge, this is the first study to propose the concept of THA and early diastole $-d\widehat{\varphi}/d\widehat{V}$ as measures of diastolic functional property and their application may provide greater insight into heart failure. Future study will perform invasive hemodynamic measurements and simultaneous echocardiography for assessing routine measures of diastolic function and compare them to the $\widehat{\varphi}\widehat{V}$ loop derived measures to comprehensively assess all the factors responsible for this phenomenon.

## Abbreviations

CMR: Cardiovascular magnetic resonance
THA: Torsion hysteresis area
LV: Left ventricle
EF: Ejection fraction
HTN: Hypertension
EDPVR: LV end diastolic pressure volume relationship
CMR: Cardiovascular magnetic resonance
BP: Blood pressure
BNP: Brain natriuretic peptide
ED: End diastole
ES: End systole
